# Disrupted Glutamate Signaling in *Drosophila* Generates Locomotor Rhythms in Constant Light

**DOI:** 10.3389/fphys.2020.00145

**Published:** 2020-03-06

**Authors:** Renata Van De Maas de Azevedo, Celia Hansen, Ko-Fan Chen, Ezio Rosato, Charalambos P. Kyriacou

**Affiliations:** ^1^Department of Genetics and Genome Biology, University of Leicester, Leicester, United Kingdom; ^2^School of Biological and Chemical Sciences, Queen Mary University of London, London, United Kingdom

**Keywords:** *Drosophila*, circadian, LL rhythmicity, screen, glutamate, locomotor, dorsal neurons

## Abstract

We have used the Cambridge Protein Trap resource (CPTI) to screen for flies whose locomotor rhythms are rhythmic in constant light (LL) as a means of identifying circadian photoreception genes. From the screen of ∼150 CPTI lines, we obtained seven hits, two of which targeted the glutamate pathway, *Got1 (Glutamate oxaloacetate transaminase 1)* and *Gs2 (Glutamine synthetase 2).* We focused on these by employing available mutants and observed that variants of these genes also showed high levels of LL rhythmicity compared with controls. It was also clear that the genetic background was important with a strong interaction observed with the common and naturally occurring *timeless (tim)* polymorphisms, *ls-tim* and *s-tim.* The less circadian photosensitive *ls-tim* allele generated high levels of LL rhythmicity in combination with *Got1* or *Gs2*, even though *ls-tim* and *s-tim* alleles do not, by themselves, generate the LL phenotype. The use of dsRNAi for both genes as well as for *Gad (Glutamic acid decarboxylase)* and the metabotropic glutamate receptor DmGluRA driven by clock gene promoters also revealed high levels of LL rhythmicity compared to controls. It is clear that the glutamate pathway is heavily implicated in circadian photoreception. TIM levels in *Got1* and *Gs2* mutants cycled and were more abundant than in controls under LL. *Got1* but not *Gs2* mutants showed diminished phase shifts to 10 min light pulses. Neurogenetic dissection of the LL rhythmic phenotype using the *gal4/gal80 UAS* bipartite system suggested that the more dorsal CRY-negative clock neurons, DNs and LNds were responsible for the LL phenotype. Immunocytochemistry using the CPTI YFP tagged insertions for the two genes revealed that the DN1s but not the DN2 and DN3s expressed Got1 and Gs2, but expression was also observed in the lateral neurons, the LNds and s-LNvs. Expression of both genes was also found in neuroglia. However, downregulation of glial *Gs2* and *Got1* using *repo-gal4* did not generate high levels of LL rhythmicity, so it is unlikely that this phenotype is mediated by glial expression. Our results suggest a model whereby the DN1s and possibly CRY-negative LNds use glutamate signaling to supress the pacemaker s-LNvs in LL.

## Introduction

The molecular basis of the *Drosophila* circadian clock has been dissected predominantly by the use of mutant screens (Axelrod et al., 2015). This forward genetics approach has identified a number of cardinal clock genes that generate interconnected feedback loops, in which two transcription factors, CLOCK (CLK) and CYCLE (CYC), play centre stage by dimerizing and activating transcription of *period (per)* and *timeless (tim)* during the subjective day (Hardin and Panda, 2013). PER and TIM begin to accumulate, but a series of posttranslational modification by kinases and phosphatases followed by degradation, delays the accumulation of PER until the late subjective night (Top et al., 2018). Then, PER-TIM enter the nucleus of clock cells and inhibit CLK/CYC, thereby negatively regulating their own *(per* and *tim)* genes. During the next subjective day, PER and TIM are degraded which releases CLK/CYC to return to the *per/tim* promoters and re-activate transcription. CLK also intersects with two other loops defined by PDP1ε/VRI and CWO which stabilize the oscillating system (Hardin and Panda, 2013).

While the clock is self-sustaining in constant conditions, it nevertheless responds to environmental stimuli, particularly light. Under a light-dark cycle, at dawn, CRYPTOCHROME (CRY), a blue light photoreceptor is activated and this leads first to the degradation of TIM (and CRY), followed by PER, and the transcription-translational cycle starts again as CLK/CYC return to the *per/tim* promoters (Stanewsky et al., 1998; Ceriani et al., 1999). The light input pathway to the clock depends not only on the photoreceptor CRY but also on the rhodopsins and the visual system (Kistenpfennig et al., 2017; Leung and Montell, 2017; Li et al., 2018; Ogueta et al., 2018). In addition, a number of other factors, both cell autonomous and non-autonomous including Jetlag, Ramshackle, Quasimodo and cell-to-cell communication are important in the degradation of TIM/CRY after light exposure (Koh et al., 2006; Peschel et al., 2006, 2009; Tang et al., 2010; Chen et al., 2011; Ozturk et al., 2013).

Neurogenetic studies of the fly clock over the past 15 years have identified a set of 150 circadian neurons in the brain divided into seven major groupings, of which the PDF-positive small ventral lateral neurons (s-LNvs) have been described as representing the pacemaker (Top and Young, 2018). However, several laboratories, including ours, have demonstrated that manipulation of any one group of clock neurons has implications for the functioning of the others, highlighting the importance of their network organization (Dissel et al., 2014; Yao and Shafer, 2014; Yao et al., 2016; Chatterjee et al., 2018; Lamba et al., 2018; Delventhal et al., 2019; Schlichting et al., 2019). We have suggested that such organization is of paramount importance in defining the properties of the clock as we have shown that the period of the clock is an emergent property of the network and not a property of any single neuron or group (Dissel et al., 2014). This suggests that other circadian properties, among which, entrainment, might result from network interactions rather than by cell-autonomous properties of clock neurons (Lamba et al., 2018).

Under constant light (LL), wild-type flies become behaviorally arrhythmic but *cry*^*b*^ and *cry*^0^ mutants maintain rhythmic locomotor cycles (Emery et al., 2000; Dolezelova et al., 2007). These results suggest that CRY plays a role not only as the dedicated circadian photoreceptor under these conditions, but also as the light gateway into the pacemaker(s) that determine rhythmic behavior, with the mutation apparently blocking all light input including that from the rhodopsins. However, light-dark cycles can still entrain *cry* mutants via the rhodopsins (reviewed in Senthilan et al., 2019). Alternatively, we could argue that such a far-reaching effect of the mutants might derive from CRY regulating the cross-talk among neurons. This hypothesis stems from the finding that light-activated CRY can directly affect neuronal firing (Fogle et al., 2011, 2015; Baik et al., 2017) and has become even more compelling after observing that the PDF-expressing neurons (including the so-called “pacemaker” neurons) are not a hub for circadian light responses. In fact retinal and sub-retinal (Hofbauer-Buchner eyelets) photoreceptors connect to and excite the majority of clock neurons via interneurons (Li et al., 2018).

A strategy to further investigate light entrainment would be to search for mutants that are rhythmic in LL (Dubruille et al., 2009). We have therefore performed such an analysis using the Cambridge Protein Trap Insertion (CPTI) lines in which a pigP (piggyback P-element) that includes YFP and affinity tags (for pulldowns and mass spectrometry) have been inserted between the coding sequences of nearly 400 genes using splice acceptor/donor site targeting (Lowe et al., 2014). The YFP motif generates an additional internal domain within the targeted protein, so it may be that some of these fusion proteins are misfolded and generate a mutant phenotype. With this in mind we screened ∼150 of these lines and report a number which show rhythmicity in LL. Further analysis of two of these lines reveals that the gene traps are located within components of the glutamate signaling pathway. We embark on a series of studies focusing on these two genes as well as other members of the pathway in order to elucidate the role of glutamate signaling in light-dependent behavioral rhythmicity.

## Materials and Methods

### Fly Stocks

All fly lines were maintained at 25°C under a light-dark cycle (LD12:12). Candidate genes were downregulated using dsRNAi crossed initially to the *tim-gal4* driver and incorporating *UAS-dicer2* into the crossing scheme to enhance the downregulation. Most of the *UAS-RNAi* lines were obtained from VDRC and available mutants for the genes of interest were obtained from the Bloomington stock centre.

### Locomotor Screening

The CPTI lines (Cambridge Protein Trap Insertions) were screened by placing 2–3 day old male flies in Trikinetics activity monitors at 25°C for 2–3 days in LD12:12 before releasing them under LL (39 μW/cm^2^) for a further 7–10 days. Locomotor activity was collected in 30 min time bins and rhythmicity was analyzed by autocorrelation and spectral analysis using the CLEAN algorithm. Rhythmic individuals required both analyses to be statistically significant (see Vanin et al., 2012).

### Phase Responses

Flies were maintained in Trikinetics monitors at 25°C in LD12:12 for 3 days. During the third night a 10 min light pulse (39 μW/cm^2^) was administered 3 h (ZT15) or 9 h (ZT21) after lights off (ZT12). The flies were kept in DD after the light pulses and locomotor behavior recorded for several days. A control group of flies of the same genotype did not receive the light pulse. Cross-correlation was used to assess the degree of phase shift by taking the locomotor data from 48 h after the light pulses i.e., the third day, and comparing each individual fly’s experimental profile against the average of the control data. This was done by shifting by one bin at a time (lag) the two sets of data against each other and calculating a correlation coefficient for each lag. The number of 30 min bins shifted that produced the maximum correlation provided the experimental phase shift, which could be either a delay or an advance. ANOVA was used to compare the phase shifts of different genotypes.

### Western Blots

Fly heads were collected in LL at CT1, 7, 13 and 19. Western blots were performed as described previously (Sandrelli et al., 2007). α-TIM antibody (gift of Francois Rouyer, Gif, Paris) raised in rat was used at a concentration of 1:2000 with α-rat as secondary (1:10,000). α-Tubulin (1:40000) was used with α-mouse (1:6000, all Sigma-Aldrich). Image J software was used to quantify the TIM bands relative to the corresponding Tubulin.

### Polymorphisms

Naturally occurring polymorphisms were studied by PCR. The *ls-tim/s-tim* variants were genotyped using a PCR strategy published previously (Tauber et al., 2007). We also examined polymorphisms in the *jetlag (jet)* gene by amplifying a 282 bp fragment of *jet* that may harbor two variants, *jet*^*C*^ and *jet^*R*^*, that both cause LL rhythmicity in the *ls-tim* genetic background (Koh et al., 2006; Peschel et al., 2006). Both variants generate an amino acid substitution (phenylalanine to isoleucine, F209I, and serine to leucine S220L). The primers used were *jet 5′-CGCGTACTCAAGCTGTCC* and *jet 3′-CACGCCATAGTCGGAGAT* at an annealing temperature of 64°C and PCR generated fragments were directly sequenced.

### Immunofluorescence

This was performed for the following genotypes: *Got1-YFP/qsm-gal4^104280^; dsRed/*+, *Gs2-YFP/qsm-gal4^104280^; dsRed/*+, *Got1-YFP/myr-RFP; repo-gal4/*+, *Gs2-YFP/myr-RFP; repo-gal4/*+. Prior to collection at the indicated ZT22, flies from different strains were entrained for at least 2 days to LD12:12 conditions. Light intensity was ∼2500 lux. Flies with indicated genotypes were fixed in 4% paraformaldehyde/PBS (3 mM NaH2PO4, 7 mM Na2HPO4, 154 mM NaCl) for 2 h at room temperature. After fixation, the samples were washed 3× by PBS and the fly brains were dissected under a microscope. The dissected brains were collected in PBS 0.1% Triton X-100 and transferred to 4% paraformaldehyde/PBS for 30 min for post dissection fixation. Fly brains were then washed 3× in PBS 0.1% Triton X-100 and blocking with 10% goat serum in 1% PBS-T was applied for 2 h before staining with guinea pig anti-PDP-1ε (1:5000, Benito et al., 2007) in 0.3% PBS-T at 4°C for 48 hr. After washing 3× with 0.1% PBS-T, the samples were incubated at 4°C overnight with anti-guinea pig antibody conjugated with fluorophore, Alexa Fluor 647 nm (Molecular Probes) diluted 1:300 in 0.3% PBS-T. Brains were washed 4× in 0.1% PBS-T and water before being mounted in Vectashield. Samples were stored at 4°C until examination under a LSM-510 META confocal microscope (Zeiss, Germany) or a Leica SP5 confocal microscope.

## Results

### Gene Traps Reveal 7 Putative Loci in the Circadian Light Input Pathway

**FIGURE 1:**
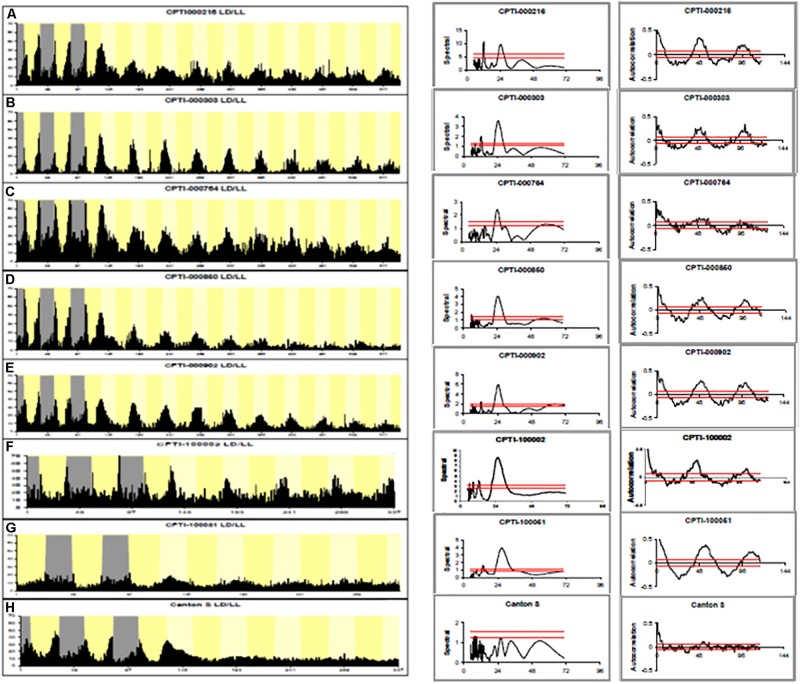
Locomotor activity rhythms in LL of CPTI genotypes. Left-hand panels. **(A–E)** 3 days LD, 10 days LL. **(F,H)** 3 days LD, 5 days LL. **(G)** 2 days LD, 5 days LL Gray: dark. Dark yellow: subjective day. Histograms are shown for each CPTI genotype that showed rhythmicity in LL as well as Canton-S controls. Each graph represents the average activity collected in 30 min time bins of all the flies per genotype so arrhythmic flies are included (see Table 1 for results and N’s). **(A)** CPTI-000216. **(B)** CPTI-000303. **(C)** CPTI-000764. **(D)** CPTI-000850. **(E)** CPTI-000902. **(F)** CPTI-100002 **(G)** CPTI-100051. **(H)** Canton S. All the fly lines were rhythmic, except Canton S **(H)**. Middle panels: Spectral analyses using CLEAN of individual activity records. The red horizontal line represent the 99% (top) and 95% (lower) confidence limits based on 1000 random permutations of the data. Right-hand panel: corresponding activity record but analyzed with autocorrelation. 95% confidence limits represented as tapered red horizontal lines.

Of 147 YFP lines screened, 7 showed rhythmicity under LL at levels between 50 and 94%. Figure 1 shows the average locomotor histograms for the 7 lines plus a control line and examples of individual spectral analyses of locomotor records. Table 1 provides the statistical analyses of these data. Each CPT line was also tested in constant darkness (DD) and all were rhythmic although the line *CPTI00051* in which the insertion lies in the *Rab11* locus also showed a significantly longer free-running period in DD (26.0 ± 0.3 h). Table 1 shows the identity of the genes trapped in each of these lines and the free-running period in LL. We then obtained the available *UAS-RNAi* lines or any mutants that existed for these seven loci. Table 2 shows that RNAi driven by *timgal4* for *Got1 (Glutamate oxaloacetate transaminase 1)* and *Gs2 (Glutamine synthetase 2)* and the available mutant males *w/Y; P(GT1)Got1/P(GT1)Got1 and wP(GT1)Gs2/Y*, confirmed the LL rhythmic phenotype observed in *Got1*^*YFP*^ and *Gs2*^*YFP*^ (Supplementary Figure S1). In the other lines either the mutants could not confirm the phenotype, or the RNAi revealed high levels of LL rhythmicity, but so did their corresponding *UAS-RNAi* parental controls, suggesting leakage and/or a background effect (Supplementary Table S1). One interesting exception was *Glycogenin (CPTI-000902)* which gave extremely high levels of LL rhythmicity for both the gene trap and the RNAi (95%) although the *UAS-RNAi* control line also generated 56% LL rhythmicity. Several of the VDRC *UAS-RNAi* lines also gave high levels of rhythmicity in LL suggesting that they carry additional genetic variant(s) within the circadian light input pathway (although not those for *Gs2* nor *Got1*, Tables 1, 2).

**TABLE 1:** Results of initial LL screen.

**\CPTI line**	**Trapped**	**% LL rhythms**	**Period**	***tim-gal4* RNAi**	**% (N)**	**Period**	**Mutants**	**% LL rhythms**	**Period**
	**gene**	**(N)**	**(sem)**	***VDRC***	**rhythmic**	**(sem)**		**(N)**	**(sem)**
000216	*lk6 kinase*	74 (31)	24.7 (0.2)	*30389*	31.8 (22)		*lk61*	12.5 (8)	–
				*32885*	28.6 (14)		*lk62*	18.8 (16)	–
				*UAS-30389*	*nd*				
000303	*Got1*	90 (20)	25.0 (0.15)	*108247*	88.8 (18)	27.6 (0.7)	*P{GT1}Got1*	84.6 (22)	29.8 (0.8)
				108247*	89.6 (29)	26.9 (0.5)	P{wHy}Got1**	0 (13)	–
				*UAS-108247*	33.4 (9)	–			
				*8340-R2*	62.5 (8)	26.5 (0.8)			
				8340-R2*	41 (22)	–			
				*8340-R1*	26 (27)	–			
				8340-R1*	43 (22)				
				*UAS-8340R-2*	8.0 (13)	–			
				*UAS-8340R-1*	9.1 (11)	–			
000764	*kat80 (katanin80)*	66.6 (21)	24.5 (0.3)	*24175*	58.3	28.2 (0.6)	*P{SUPor-P}kat80*	21.6 (26)	–
				*UAS-24175*	55 (11)	28.8 (1.9)	*P{EP}kat80*	0 (15)	–
000850	*Pbl (pebble)*	69.2(26	25.5 (0.4)	*35349*	14.3 (7)	–	*pbl^3^/+*	50 (15)	25.5 (0.9)
				*35350*	78.5 (28)	26.4 (0.3)	*pbl5/+*	12.5 (16)	–
				*UAS-3530*	73	26.2 (0.5)			
000902	*Glycogenin*	94.7 (19)	24.7 (0.1)	*35452*	93.7 (6)	28.9 (0.8)			
				*UAS-3530*	56.3 (55)	28.8 (0.6)			
100002	*Gs2*	50 (18)	25.1 (0.2)	*32929*	81.3 (16)	25.4 (0.5)	*P{GT1}Gs2*	80 (25)	28.6 (0.6)
				3929*	80.6 (31)	26.0 (0.3)			
				*UAS-32929*	15 (53)	–			
100051	*Rab11*	69 (9)	26.3 (0.8)	*22198*	*nd*	*nd*	*P{wHy}Rab11*	31 (16)	–

*including UAS-dicer2 **P(wHy)Got1 was also poorly rhythmic in DD (35%).

**TABLE 2:** Effects of mutations or *timgal4-*driven downregulation on LL rhythmicity.

**Genotype (id)**	**% LL rhythms**	**N**	***tim* alleles**
***A. Mutants on w^1118^ background (F2)***			
*w^1118^/Y; P{GT1}Got1/P{GT1}Got1*	61	32	*ls-tim,s-tim, ls/s-tim*
*w^1118^/Y; P{GT1}Got1/+*	44	31	*ls-tim,s-tim, ls/s-tim*
*w^1118^/Y*	26	31	*ls-tim,s-tim, ls/s-tim*
*wP{GT1}Gs2/Y*	63	31	*ls-tim,s-tim, ls/s-tim*
*w^1118^/Y*	31	29	*ls-tim,s-tim, ls/s-tim*
*wP{GT1}Gs2/Y*	21.8	32	*s-tim*
*w/Y; P{GT1}Got1*	20	32	*s-tim*
***B. RNAi***			
*w/Y; tim-gal4/+; Gs2RNAi/+* (32929)	81.3	16	*ls-tim,s-tim, ls/s-tim*
*UAS 32929*	15	53	*ls-tim, s-tim. ls/s-tim*
*w/Y; tim-gal4/+*	4	51	*s-tim*
*w/Y; tim-gal4/Got1RNAi (UAS VDRC 108247)*	72.2	18	*ls/s-tim*
*w/Y; tim-gal4/+; Got1RNAi/+ (8340R-2)*	37.5	8	*s-tim*
*w/Y; tim-gal4/Got1RNAi (8340R-1)*	26	27	*s-tim*
*UAS VDRC 108247*	33.4	9	*ls-tim*
*UAS 8340R-1*	9	11	*s-tim*
*UAS 8340R-2*	8	13	*s-tim*
***C. P(GT1)* insertion controls**			
*w1118/Y; P{GT1}Tom7BG02496 (12698)*	*21.9*	32	*ls-tim*
*w1118/Y; P{GT1}AdhAdhrBG01049 (12535)*	*20*	30	*ls-tim*
*w1118/Y; P{GT1}prtpBG00450 (12488)*	*16.2*	31	*ls-tim*

*The *tim* allelic background for each group is also shown.*

### Genetic Background Interacts With *Got1* and *Gs2* Mutations to Generate LL Rhythmicity

As both *Got1* and *Gs2* are involved in glutamate metabolism, we focused on these genes for the rest of the study. Mutations in both genes involved the *w;P(GT1)* element. To test whether the *w;P(GT1)* background was sensitized for LL rhythms, we examined another three *w;P(GT1)* insertions in *Tom7, AdhAdhr*, and *prtp.* All three *w;P(GT1)* lines gave low levels of rhythmicity in LL, 22, 20, and 16% respectively (Table 2). Consequently the genetic background that provided the *w;P(GT1)* screen was not of itself responsible for the high levels of LL rhythmicity in *Got1* and *Gs2.*

We also crossed the *Got1 w;P(GT1)* mutant females to *w*^1118^ (white-eyed) mutant males and then assessed the LL rhythmicity in the F2 generation where we could distinguish by eye color the mutant homozygotes, heterozygotes and the white-eyed wild-type. We observed 61% LL rhythmicity in the mutant homozygotes, 44% in heterozygotes (mean periods were 28–29 h) and considerably less LL rhythmicity in the wild-type (Table 2). When we performed a similar cross to the sex-linked *Gs2 (wPGT1)* mutant we observed 63% LL rhythmicity in hemizygous mutant males and 31% rhythmicity in the wild-type white-eyed F2 males (Table 2). The LL rhythmicity of both *Gs2* and *Got1* mutants after randomizing the genetic background in this way was ∼20% less than the ∼80% we observed with the original YFP gene traps (Table 1), suggesting further polymorphisms that were enhancing or reducing the LL effects.

### *timeless* but Not *jetlag* Polymorphisms Interact With *Got1* and *Gs2* Mutations to Generate Enhanced Levels of LL Rhythmicity

The best known polymorphism in the light input pathway involves the naturally occurring *s-tim/ls-tim* variant, in which the *ls-tim* allele reduces circadian photosensitivity (Sandrelli et al., 2007; Tauber et al., 2007). We therefore addressed the *tim* status of the w;*P(GT1)* lines and *w*^1118^ using PCR. The original *Gs2*^*YFP*^ and *Got1*^*YFP*^ alleles whose results are shown in Table 1 are in the *ls-tim* background, whereas *w*^1118^ carries *s-tim.* Consequently, the decrease in levels of LL rhythmicity in the F2 crosses with *w*^1118^ could be due, in part, to the segregating *ls-tim/s-tim* variant (Table 2). We repeated the crosses of each mutant to *w*^1118^ and inspected the *ls-tim/s-tim* status of each fly after it had also been investigated for LL locomotor rhythmicity. Table 3 reveals that for both genes, LL rhythmicity is associated with either *ls-tim* homo- or heterozygosity. Almost all the *ls-tim* homozygotes and half the heterozygotes are LL rhythmic. For *Gs2*, 5 homozygous *s-tim* individuals were arrhythmic in LL. Unfortunately we did not obtain any *s-tim* homozygotes for *Got1.* Based on these results it was necessary to verify the *tim* status of the three “control” *w;P(GT1)* induced mutants *Tom7, AdhAdhr, and prtp* which did not show LL rhythmicity. Their *tim* genotyping by PCR revealed that they were all *ls-tim* homozygous (Table 2). Consequently the *ls-tim* polymorphism does not by itself cause high levels of LL rhythmicity, supporting previous studies (Sandrelli et al., 2007), yet it does enhance the LL rhythmicity of the *Gs2* and *Got1 w;P(GT1)* variants.

**TABLE 3:** Segregation analysis in F2 generation for *Gs2* and *Got1* mutants crossed to *w^1118^.*

	**N**	**n^R^**	**n^AR^**
***wP{GT1}Gs2/Y***			
*ls-tim/ls/tim*	9	9	0
*ls-tim/s-tim*	16	10	6
*s-tim/s-tim*	5	0	5
***w:P{GT1}Got1/P{GT1}Got1***			
*ls-tim/ls/tim*	14	12	2
*ls-tim/s-tim*	17	7	10
*s-tim/s-tim*	0	0	0

*n^*R*^, n^*AR*^ represents the numbers rhythmic or arrhythmic in LL respectively. N is total number for each *tim* genotype.*

The behavior of w;*P(GT1)* mutants for *Gs2* and *Got1* resembles that of *Veela* flies which carry the *jetlag* variant *jet^*c*^*, and are rhythmic in LL only in the presence of the less light-sensitive LS-TIM isoform (Peschel et al., 2006). We therefore investigated the *Gs2, Got1* and *w*^1118^ mutants for the presence of *jet*^*c*^ or the rare allele, *jet^*r*^.* PCR of a 282 *jet* bp fragment that includes both variant sites followed by sequencing did not reveal any polymorphism (data not shown).

We then genotyped the other lines for *Got1* and *Gs2* as well as the parental RNAi lines for the *tim* and *jet* variants. Table 2 shows that the flies that were downregulated for *Gs2* with *timGal4 (s-tim)* would segregate both *tim* alleles as *UAS 32929* was polymorphic, yet they gave >80% LL rhythmicity. The downregulated lines for *Got1* were heterozygous *s-tim/ls-tim* or *s-tim* homozygous (Table 2). Clearly having at least one copy of *ls-tim* enhances LL rhythmicity, but UAS control flies that are *ls-tim* homozygotes do not give high levels of LL rhythmicity (see line *108247*) so the very high levels we observe with *Gs2* and *Got1* variants represent these alleles interacting with *tim.* None of these lines were polymorphic for the *jet* alleles.

We also placed the *Gs2* and *Got1* alleles on the *s-tim* background by crossing to *w*^1118^ and generating *s-tim* homozygous lines for *Gs2* and *Got1* from the F2 generation using PCR. We observed that on this genetic background, only 20 and 21% respectively of the males were rhythmic in LL, further underscoring the interaction of these two genes with the *ls*-*tim* polymorphism (Table 2).

### Under LL Conditions *Got1* and *Gs2* Mutants Show High Amplitude TIM Cycling and More Abundant TIM Than Wild-Type

**FIGURE 2:**
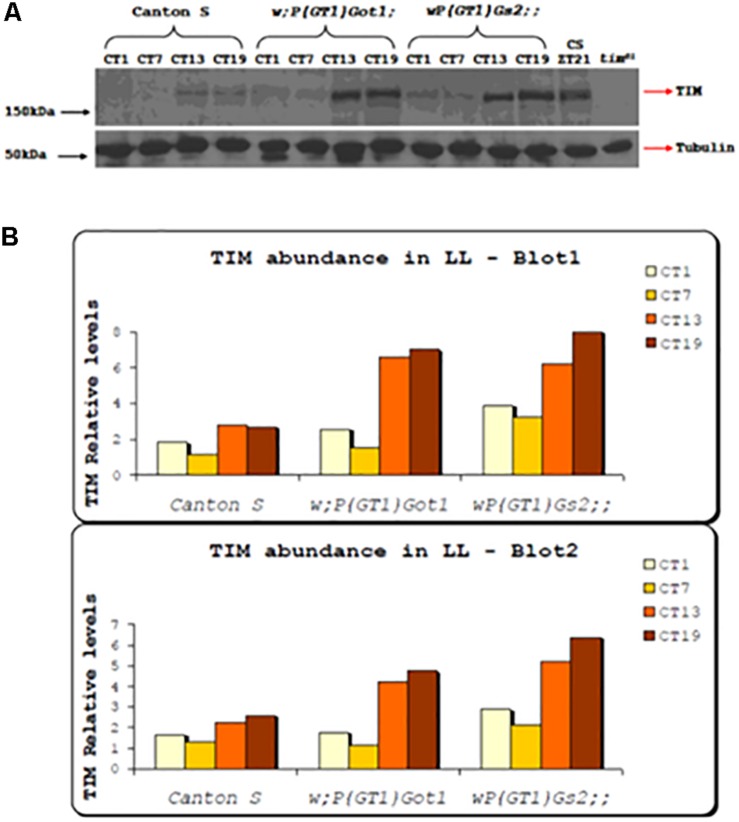
TIM expression in *Got1* and *Gs2* mutants. **(A)** Western blot from head extract of *w;P(GT)Got1, wP(GT1)Gs2* and Canton-S flies collected in LL. Canton-S collected in LD cycles and *tim*^01^ mutants were used as positive and negative controls respectively. Genotypes and time points for collection (CT) are indicated above the blot. **(B)** Quantitative analysis using Image J of two replicate blots.

Western blots were performed from fly heads for the *w;P(GT1)Got1* and *wP(GT1)Gs2* mutants and compared to Canton-S. Levels of TIM were compared with tubulin under free running LL conditions for four time points, CT1, 7, 13, and 19. Two replicate blots were performed and both gave almost identical results (Figure 2). Levels of TIM compared to tubulin cycled with a 3–5 fold peak-to-trough amplitude for both *Got1* and *Gs2* mutants, with a clear peak at CT19 whereas in wild-type the relative amplitude was blunted to 1.5–2 fold with a peak at either CT13 or CT19. Furthermore TIM was approximately 2–4 times as abundant in the mutants compared to wild-type under LL and very similar to wild-type flies maintained in LD cycles (Figure 2). These results reinforce the view that under LL the two mutants block the normal light-induced degradation of TIM (Myers et al., 1996) but nevertheless maintain high amplitude TIM cycling.

### *Got1* Mutants Reduce Circadian Locomotor Responses to Brief Light Pulses

**FIGURE 3:**
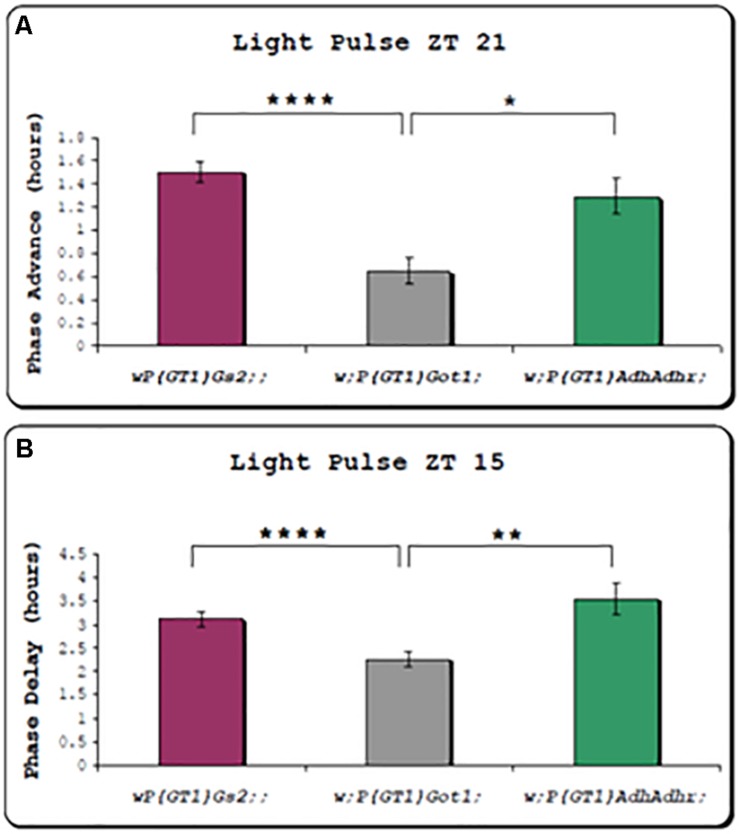
Circadian phase responses of locomotor rhythms to brief light pulses for *Got1* and *Gs2* mutants. All three mutants are on the *ls-tim* background. Two replicate experiments were performed for the Got1 and Gs2 lines, and one for the control line. At ZT21 *Got1 n* = 58, *Gs2 n* = 60, *Adh n* = 23. At ZT15 *Got1 n* = 63, *Gs2 n* = 61, *Adh n* = 11. **(A)** Phase advances (± sem) to a light pulse at ZT21 **(B)** Phase delays to a light pulse at ZT15 *p < 0.05 **p < 0.01 ****p < 0.0001.

We also examined the effects of 10 min light pulses on the phase of the locomotor cycle in the two *w;PGT1 Got1* and *Gs2* mutants and compared them to the *w;P(GT1)AdhAdhr* mutant as a control. We observed that light pulses at ZT15 (3 h after lights off in a LD12:12 cycle) delayed the clock by 3 to 3.5 h for *Gs2* and *AdhAdhr*, whereas the phase delay for *Got1* was significantly reduced to 2 h (Figure 3). Similarly, the light pulse at ZT21 late at night caused an advance of 1.2–1.4 h for *Gs2* and *AdhAdhr*, whereas this was again significantly reduced to 0.6 h for *Got1.* All these lines are in the less light-sensitive *ls-tim* background (Sandrelli et al., 2007); nevertheless, the smaller phase shifts under these conditions appear to be specific to *Got1.*

### Anatomical Dissection of Clock Neurons Mediating LL Locomotor Rhythmicity

**FIGURE 4:**
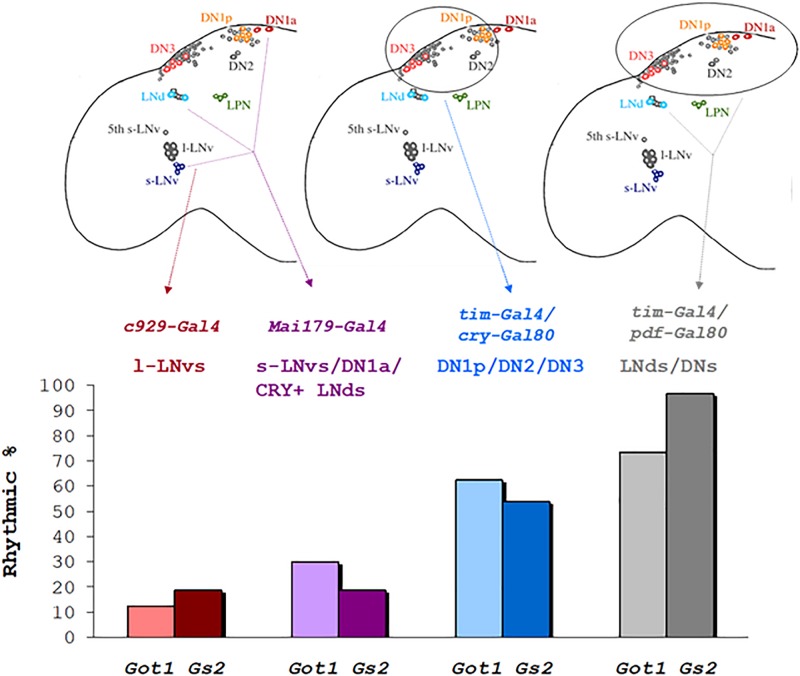
Rhythmicity in LL after downregulation of *Got1* and *Gs2* in different neuronal clock clusters % rhythmicity in LL is represented when downregulation of *Gs2* or *Got1* has been applied to specific groups of clock neurons using the corresponding *gal4/gal80* drivers.

We attempted to define the relevant clock neurons that were mediating the LL rhythmicity of *Got1* and Gs2 mutants. We therefore used the *UAS-RNAi* constructs to downregulate each gene’s expression in different clock neuronal clusters. Knockdown in the peptidergic neurons including the l-LNvs but excluding other clock neurons using the *c929-Gal4* driver resulted in arrhythmicity in LL, thus excluding glutamate from within the l-LNvs from maintaining rhythms in LL (Table 4). Similarly the great majority of flies were arrhythmic in LL when the *mai79-gal4* driver was used, which drives expression in the s-LNvs and the three CRY-positive LNds and possibly one of the two DN1a neurons (Grima et al., 2004). When we combined *tim-gal4* with *cry-gal80* and targeted expression to the DN1p, DN2, and DN3, and three LNd CRY-negative cells in the dorsal region, *Got1* downregulation generated 62.5% rhythmicity in LL and 53% for *Gs2.* We also combined *tim-gal4* with *Pdf-gal80* and restricted downregulation to all the LNds and DNs. In these cases a much higher level of 73% of flies were rhythmic in LL for *Got1* and a remarkable 90% were rhythmic for *Gs2.* This was in spite of the flies segregating the s-*tim* variant. The parental controls for these crosses were considerably less rhythmic in LL than in the experimental flies (Table 4). Figure 4 illustrates the correlation between LL rhythmicity and anatomical expression for the two downregulated genes. It is clear that the DNs play a major role, particularly those that are CRY-negative but with another significant component arising from the CRY-negative and possibly CRY-positive LNds.

**TABLE 4:** Anatomical dissection of glutamate-mediated LL rhythmicity and *tim* background genotype.

***Genotype (VDRC)***	**% LL rhythms**	**N**	***tim* alleles**
***A. Got1, Gs2***			
*w/Y; c929-gal4/Got1 RNAi (108247)*	12.5	32	*ls/s-tim*
*w/Y; c929-gal4/+; Gs2 RNAi/+ (32929)*	18.7	32	*ls/s-tim, ls-tim, s-tim*
*w/Y; mai79-gal4/Got1 RNAi*	30	30	*ls/s-tim*
*w/Y; mai79-gal4/+; Gs2 RNAi/+*	18.8	32	*ls/s-tim, ls-tim, s-tim*
*yw/Y; timGal4/Got1 RNAi; cry-gal80/+*	62.5	20	*ls/s-tim*
*yw/Y; timGal4/+; cry-gal80/Gs2 RNAi*	53	32	*ls/s-tim, ls-tim, s-tim*
*yw/Y; timGal4 Pdf-gal80/Got 1 RNAi; Pdf-gal80/+*	73.3	30	*ls/s-tim, ls-tim, s-tim*
*yw/Y; timGal4 Pdf-gal80/+;Pdf-gal80/Gs2 RNAi*	96	30	*ls/s-tim, ls-tim, s-tim*
*w/Y; c929-Gal4/+*	0	31	*s-tim*
*yw/Y; tim-gal4/+;cry-gal80/+*	28	32	*ls/s-tim, ls-tim, s-tim*
*yw/Y; tim-gal4 Pdf-gal80/+; Pdf-gal80/+*	41	32	*ls/s-tim, ls-tim, s-tim*
*w/Y mai79-gal4/+*	15.7	19	*s-tim*
***B. glutamate receptor, GAD***			
*w/Y;tim-gal4/+; DmGluRA RNAi/+ (1793)*	52	31	*ls/s-tim*
*w/Y;tim-gal4/+; DmGluRA RNAi/+ (1794)*	65.6	32	*ls/s-tim*
*w/Y;tim-gal4/+; DmGluRA RNAi/+ (103736)*	64.5	31	*ls/s-tim*
*w/Y;tim-gal4/+; Gad1 RNAi/+ (32344)*	56.3	32	*ls/s-tim, ls-tim, s-tim*
*UAS DmGluRA RNAi/+ (1793)*	26.7	30	*ls-tim*
*UAS DmGluRA RNAi/+ (1794)*	25	32	*ls-tim*
*UAS DmGluRA RNAi/+ (103736)*	15.6	32	*ls-tim*
*UAS Gad1 RNAi/+ (32344)*	31.3	30	*ls/s-tim, ls-tim, s-tim*
*w/Y; tim-gal4*	4	51	*s-tim*
***C. repo-gal4***			
*Got1 RNAi/+; repo-gal4/+;*	27	26	*s-tim ?*
*yw/Y; timGal4/Got1 RNAi;*	68	25	*s-tim*
*Gs2RNAi/repo-gal4:*	29	21	*ls-tim,s-tim, ls/s-tim*
*yw/Y; timGal4 /+;+ /Gs2 RNAi*	66	31	*ls-tim,s-tim, ls/s-tim*

We extended our analysis to include downregulation of the metabotropic glutamate receptor DmGluRA. We used three different VDRC RNAi lines and crossed them to *tim-gal4.* All three lines gave >50% rhythmicity in LL with ∼24 h periods (Table 4). We did not, however, observe any lengthening of period in these knockdowns in DD (Supplementary Table S2) as reported by Hamasaka et al. (2007), even though we used three independent *UAS-RNAi* lines. Glutamate serves as the precursor for the synthesis of the inhibitory GABA neurotransmitter and this reaction is catalyzed by glutamate decarboxylase (GAD). We downregulated *Gad* using *tim-gal4* and observed a high level of LL rhythmicity. The parental controls for all these manipulations were considerably less rhythmic under LL (Table 4). We genotyped the *UAS-RNAi* lines, *DmGluRA* and *Gad* for *tim* and *jet* polymorphisms. All *UAS-DmGluRA* lines were homozygous *ls-tim*, whereas *UAS-Gad* RNAi lines were polymorphic and showed all three *tim* genotypes. All lines were monomorphic for wild-type *jet.* The *w:tim-Gal-4* line they were crossed to was homozygous *s-tim.* Therefore the experimental flies with *UAS-DmGluRA* were heterozygous (*ls/s-tim*) whereas those with *UAS-Gad* were either heterozygous or homozygous for both *tim* alleles (Table 4).

### Spatial Distribution of Got1 and Gs2 Positive Cells Among Clock Neurons

**FIGURE 5:**
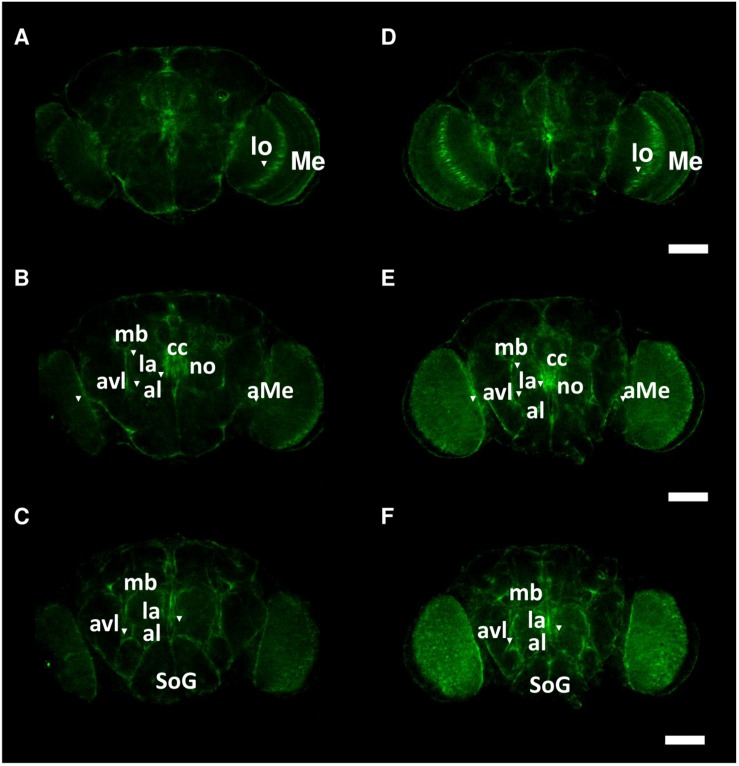
Spatial patterns of Got1-YFP and Gs2-YFP expression in the fly brain. Frontal views of anterior **(A,D)**, intermediate **(B,E)** and posterior parts **(C,F)** of brains are shown for Got1-YFP **(A–C)** and Gs2-YFP **(D–F)**. Inverted triangles mark the strong expression foci of YFP signals around following anatomical structures: aMe: accessory medulla, al: antennal lobe, avl: anterior ventral lateral protocerebrum, cc: central complex, la: lateral accessory lobe, lo: lobula, mb: mushroom body, Me: medulla, SoG: suboesophagus ganglion. Scale bars: 75 μm. magnification: 10×. Nine Gs2-YFP and 11 Got1-YFP brains were investigated. The intensity of expression between Gs2-YFP and Got1-YFP were not investigated.

**FIGURE 6:**
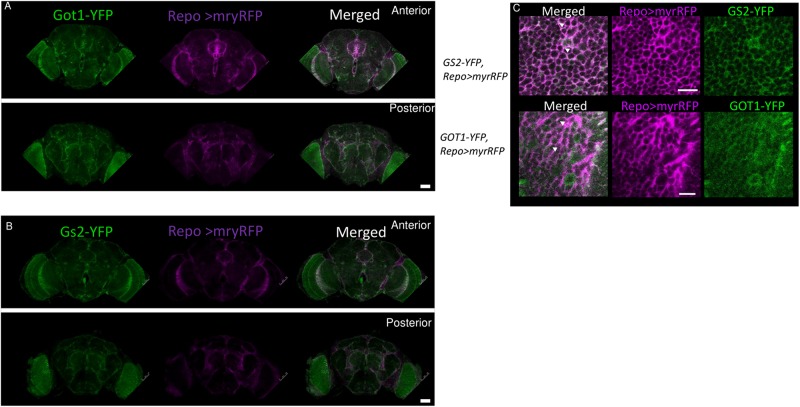
Got1-YFP and Gs2-YFP expression in glial cells in the fly brain. Frontal views of anterior and posterior parts the fly brains are shown for Got1-YFP **(A)** and Gs2-YFP **(B)**. *repo*>myrRFP marks the cell membranes of glial cells (magenta), which overlap with both Got1-YFP and Gs2-YFP signal (green) in optic lobes and inter-neuropils (white areas in merged section). Scale bars: 50 μm. Magnification: 10×. Nine Gs2-YFP and seven1 Got1-YFP brains were investigated. **(C)** Detailed overlaps between GS2-YFP and GOT1-YFP (green) and repo-gal4 driven myrRFP (magenta). An example image at inter-neuropil regions are shown. Similar patterns are detected between YFP and RFP signals. White areas and arrowheads indicate examples of overlaps between the YFP and RFP signals at glial membranes. Scale bars: 10 μm. Magnification: 40×.

To investigate the spatial distribution of Got1 and Gs2 in the fly brain, we examined the endogenous YFP signal in the gene-trap *Got1^*YFP*^/* + and *Gs2^ YFP^/* + flies. Overall the Got1 and Gs2 derived YFP expression patterns are ubiquitous in various areas between neuropils and in the brain cortex (e.g., around medulla, lobula, and mushroom body, Figure 5). This result is consistent with the earlier high throughput study including both YFP trap lines (Knowles-Barley et al., 2010). We did not detected any gross spatial differences of expression between Got1-YFP and Gs2-YFP. Notably the expression patterns for Got1-YFP and Gs2-YFP appeared to include glial cells. To confirm this observation, we examined overlaps between YFP and the glial reporter *repo-gal4* (Awasaki et al., 2008) by driving myr-RFP (membrane tethered RFP by myristoylation signal fusion, from Henry Chang). Consistently, we found Got1 and Gs2 positive cells overlapping with glial cells in brain cortex and between neuropils (Figures 6A–C).

**FIGURE 7:**
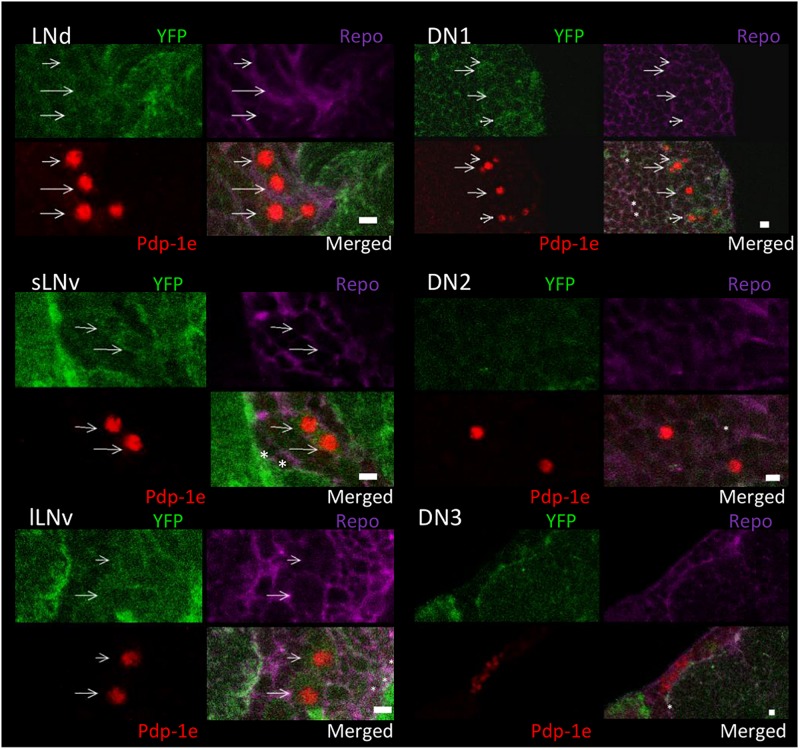
Got1-YFP expression in clock neurons in the fly brain. Four panels of each clock neuronal group are shown, Pdp-1ε (red) indicates clock neuron nucleus. Repo >myrRFP marks the cell membranes of glial cells (magenta). Individual cell containing both cytosolic Got1-YFP signal (green) and Pdp-1ε nuclear staining are detected in LNs and DN1s and are indicated by arrows. Asterisks indicate the example of overlaps between glial cells and YFP signals. Scale bars: 4 μm. Magnification: 40×. Five brains were investigated. Single optical slides are shown with dorsal on the top position.

**FIGURE 8:**
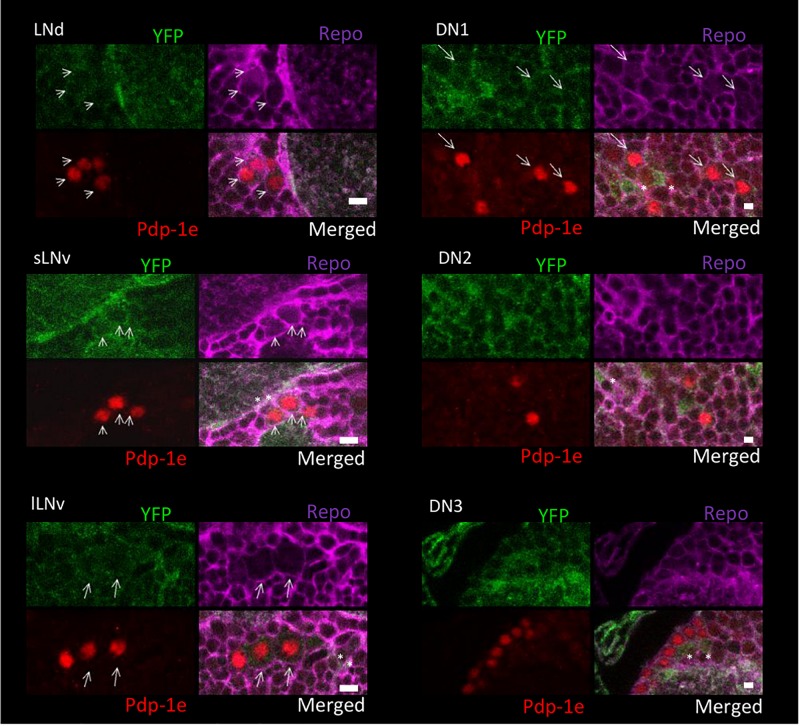
Gs2-YFP expression in clock neurons in the fly brain. Four panels of each clock neuronal group are shown, Pdp-1ε (red) indicates clock neuron nucleus. *Repo* >myrRFP marks the cell membranes of glial cells (magenta). Individual cell containing both cytosolic Gs2-YFP signal (green) and Pdp-1ε nuclear staining are detected in LNs and DN1s and are indicated by arrows. Asterisks indicate the example of overlaps between glial cells and YFP signals. Scale bars: 4 μm. Magnification: 40×. Four brains were investigated. Single optical slides are shown with dorsal on the left-top position.

**FIGURE 9:**
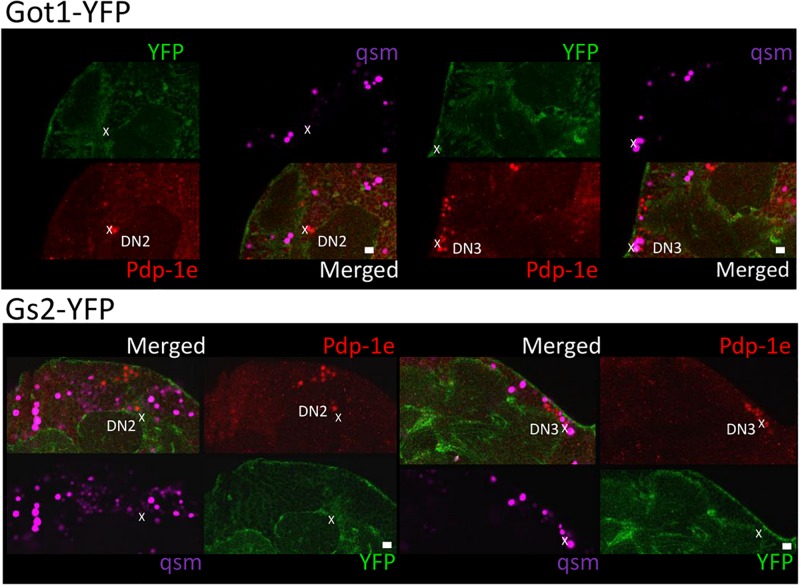
Gs2-YFP and Got1-YFP surround qsm + DNs in the fly brain. Four panels of each brain area close to DN2 and DN3 are shown for Got1-YFP **(upper panel)** and Gs2-YFP **(lower panel)**. Pdp-1ε (red) indicates clock neuron nuclei. *qsm^104^-gal4* >dsRED marks qsm + dorsal cells (qsm, magenta). Individual cell containing both dsRED and Pdp-1ε nuclear staining are qsm + DN2-3s and are indicated by “x.” YFP signal surround qsm + DN2-3s but no clear overlaps could be detected. Scale bars: 10 μm. Magnification: 40×. Four brains were investigated. Single optical slides are shown with dorsal on the left-top position.

To explore the relationship among YFP positive cells and clock neurons, we applied an antibody against PDP1ε as a nuclear marker for clock neurons (Benito et al., 2007). Both YFP signals were detected peripherally to the cell bodies of the PDP1ε positive neuron, as well as in cells near clock neurons (Figures 7, 8). Within clock neurons, we detected clear cytoplasmic YFP signals in LNvs, LNds, and DN1s (Figures 7, 8). Although the behavioral data suggested that CRY-negative dorsal clock neurons including half of DN1s, DN2s and most DN3s may be responsible for the LL phenotype, we did not detect clear cytoplasmic YFP signals in DN2s and DN3s under higher magnification (Figures 7, 8). We did observe YFP signal surrounding quasimodo (qsm +) expressing DN2s and DN3s (“×” in Figure 9 and (Chen et al., 2011). Pericellular YFP signals regularly overlapped with *repo*>*myrRFP* (asterisks in Figures 7, 8), suggesting these signals could be derived from glial cell processes which were previously identified to surround neurons (Awasaki et al., 2008; Ng et al., 2011). Taken together with the behavioral results, these data imply that the LL rhythms observed in *Got1*^*YFP*^ and *Gs2*^*YFP*^ flies may be derived from abnormal glutamate metabolism in CRY-negative DN1s, the LNds and/or glial cells.

Finally, in order to investigate glial contributions to the LL phenotype we downregulated *Got1* and *Gs2* in glia using *repo-Gal4* with *timgal4* driven RNAi as controls. We observed that entrainment in LD cycles was normal, but most of the *repo-gal4* flies for both *Gs2* and *Got1* knockdown became arrhythmic almost immediately in LL, whereas flies carrying *tim-gal4* crossed to the same *UAS RNAi* constructs were considerably more rhythmic in LL (Table 4). We conclude that the LL rhythmic phenotype is predominantly caused by neuronal rather than glial glutamate signaling.

## Discussion

**FIGURE 10:**
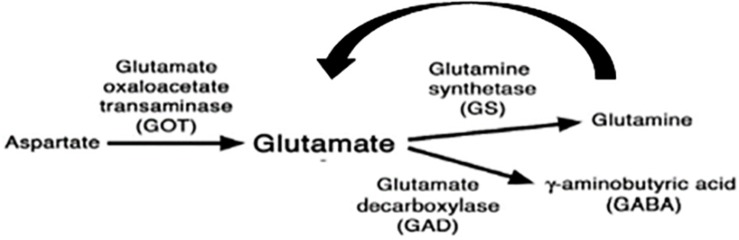
Enzymes involved in glutamate metabolism (modified from Featherstone et al., 2002).

The protein trap screen was original intended to generate lines in which the normal spatial expression of a gene could be determined with YFP and followed up by proteomic analyses using the incorporated tags. We decided to use it as a mutational screen reasoning that the addition of the YFP domain within a protein may alter its conformation and generate behavioral phenotypes. While this was initially an article of faith, it seems to have been supported by the identification of several loci that may contribute to the processing of light input into the circadian clock. We notice that all genes we identified are functional in the nervous system but are not implicated in protein degradation. One of these, the kinase encoding *lk6*, was also identified in an overexpression screen for LL rhythmicity using *EP* elements (Dubruille et al., 2009). Heterozygous *lk6/* + individuals did not give high levels of LL rhythmicity and knockdown using *tim-gal4* gave a moderate ∼30% rhythmicity (Supplementary Table S1). It may be that the YFP insertion cassette led to a more stable Lk6 product which would imitate an overexpression phenotype. However, the two most interesting genes were, *Got1* and *Gs2*, because they implicated glutamatergic signaling (Figure 10). The initial gene trap results for these two genes were supported with independently generated mutants in these genes as well as dsRNAi knockdown. The only mutation that did not give a similar phenotype was *P(wHy)Got1*, but this variant also showed a very low proportion of weakly rhythmic individuals in DD.

*tim* and *jet* polymorphisms can dramatically alter circadian photo-responsiveness (Koh et al., 2006; Peschel et al., 2006; Sandrelli et al., 2007; Tauber et al., 2007) with the *jet*^*C*^ variant interacting with *ls-tim* to generate LL rhythmicity at high levels (Peschel et al., 2006, 2009). We studied polymorphisms at both loci and it was clear that the *ls-tim* allele interacted with *P{GT1} Got1* and *Gs2* variants to enhance LL rhythmicity. However, by itself *ls-tim* was not sufficient for high levels of LL rhythmicity because several *P{GT1}* mutants homozygous for *ls-tim* were arrhythmic in LL (see also Sandrelli et al., 2007). Nevertheless, our results underline how important it is to identify these common *tim* genetic variants in the genetic background of any LL screen or indeed in any analysis of circadian photo-responsiveness.

In spite of the compelling LL phenotypes with both *Got1* and *Gs2* mutants, when the circadian clock was probed with brief 10 min light pulses, only the *Got1* mutant showed a compromised phase response in both advance and delay zones, suggesting that it is less sensitive to light than *Gs2* mutants under these conditions. This difference was not reflected under the more stringent environment of LL as the levels of rhythmicity for both mutants when on the same *tim* genetic background were very similar (Tables 1, 2), as was the enhanced stability/levels of TIM in the two mutants under LL (Figure 2). The latter result from the western blots of fly heads is intriguing because the level of TIM cycling in the mutants in LL was similar to that of wild-type in DD. This in turn suggests that the eyes of the mutants are still cycling for TIM in LL, implying that the TIM status of the dorsal clock neurons (see below) is driving the same TIM pattern in the eye. Alternatively, glutamate signaling has been reported in the eye (Kolodziejczyk et al., 2008; Richter et al., 2018) and this would also be expected to be compromised in the mutants. How this might disrupt the normal TIM cycle damping in the eye under LL is open for speculation.

In *Drosophila*, neurotransmission by glutamate is mediated by ionotropic receptors that form cation/anion channels (Dingledine et al., 1999; Anwyl, 2009; McCarthy et al., 2011) and metabotropic G-protein coupled receptors (Bogdanik et al., 2004). Previous work has implicated glutamate and its metabotropic receptor, DmGluRA, in the *Drosophila* clock circuitry (Hamasaka et al., 2007). Transgenic flies with altered expression of DmGluRA in the LNvs showed altered locomotor activity under LD and DD with a modest lengthening of the free-running (DD) period by 0.3 to 0.6 h observed with *Pdf, cry* and *timgal4* drivers. In LD cycles a strong increase in the activity after lights-off was also noticed (Hamasaka et al., 2007). However, knockdown of the receptor did not affect DD rhythms in our study using three independent *UAS-RNAi* constructs. Similarly, Collins et al. (2012) observed that reducing presynaptic glutamate levels by overexpressing Gad1 with the *timgal4; Pdfgal80* or *timgal4; crygal80* drivers had little effect on the locomotor period in DD but had an effect on the robustness of the cycle, with the former showing reduced power compared to the latter (Collins et al., 2012). This implied that the non-sLNv CRY + expressing neurons (including the DNs and LNds) were releasing glutamate and contributing to robust rhythmicity in DD.

Knockdown of the receptor *DmGluRA* using *timgal4* in our experiments led to high levels of LL rhythmicity compared to controls, further revealing the association between glutamate and circadian photo-responsiveness. Furthermore, knockdown of *Glutamate decarboxylase (Gad)* using *timgal4* also generated LL rhythmicity. The distribution of GABA, the major inhibitory neurotransmitter produced in *Drosophila* neurons, has been previously mapped to different areas of the brain (Kuppers et al., 2003). Although clock neurons do not appear to express GABA (Dahdal et al., 2010) application of GABA antisera and the use of flies expressing GFP driven by the *Gad1* promoter revealed that s-LNvs receive GABAergic inputs and utilize GABA as a slow inhibitory neurotransmitter (Hamasaka et al., 2005). DN1s and DN3s are glutamatergic, since these cells were immunolabeled for vesicular glutamate transporter (DvGluT) (Hamasaka et al., 2007). Additionally, antiserum against DmGluRA labeled the LNvs dendrites, indicating that the glutamate signal from the DNs modulates the behavior of the LNvs (Hamasaka et al., 2007). Indeed, axons from DN3s may communicate with the LNvs (Veleri et al., 2003) whereas axons from DN1s may contact the s-LNvs (Guo et al., 2016). Our neurogenetic dissection suggests that the normal arrhythmic response to LL is mediated by glutamate signaling from the DNs to the s-LNvs. The fact that *Gad1* RNAi driven by *tim-gal4* also leads to enhanced LL rhythmicity (Table 4) suggests that other GABA producing neurons [*timgal4* is broadly expressed beyond just the canonical clock neurons (Kaneko and Hall, 2000)] are nevertheless communicating with them and are important for normal photoresponsiveness.

Previous studies have also implicated the role of the DN1s, LNds, and DN3s in mediating LL behavioral rhythmicity using various neurogenetic manipulations (Murad et al., 2007; Picot et al., 2007; Stoleru et al., 2007). In particular, Murad et al. (2007) showed how *per* overexpression in clock neurons driven by *timgal4* (but not *crygal4 or Pdfgal4*) led to LL rhythmicity with the additional key observation that molecular rhythms were observed only in a subset of DN1s (Murad et al., 2007). In spite of considerable speculation in this study, the mechanism by which DN1s could escape the influence of the molecularly arrhythmic sLNvs under LL was not clear. Our study would also suggest that in *Got1*^*YFP*^ and *Gs2*^*YFP*^ and the other mutants we have used, the DN1s would be similarly liberated from the influence of the sLNvs in LL, which are likely to be molecularly arrhythmic. How compromised inhibitory glutamate signaling from DNs to the sLNvs might generate the LL TIM rhythms that we observe is difficult to explain. Nevertheless, our results are consistent with the DN1s generating the LL rhythmicity because it is maintained in *timgal4;crygal80* and *timgal4;Pdfgal80* driven flies (Figure 4). Furthermore the relatively low levels of LL rhythmicity we observed with *maigal4* driving *Got1* and *Gs2* RNAi, largely excludes the three strongly CRY-positive LNds, as well as those in the LNv cluster (also from the use of *timgal4;Pdfgal80).* The DN1a neurons may communicate through their connection with the s-LNv fibers in the dorsal brain and accessory medulla (Shafer et al., 2006; Helfrich-Forster et al., 2007).

While these other studies have been performed without identification of the *tim* background, which is very likely to have modulated their results, a consensus appears to be growing that glutamate signaling from DNs to s-LNvs may provide the network mechanism that lies at the root of the arrhythmia observed in LL. However, further proof will require the use of more refined genetic and molecular tools that identify inter-neuronal contacts as well as revealing the activities and the direction of flow of information among the clock neurons themselves as well as with associated GABA pathways.

## Data Availability Statement

All datasets generated for this study are included in the article/Supplementary Material.

## Author Contributions

CK and ER designed and supervised the study. RA and CH performed the experiments. K-FC performed the ICC. CK wrote the first draft. All authors contributed to the final manuscript. CK and ER obtained funding for the work.

## Conflict of Interest

The authors declare that the research was conducted in the absence of any commercial or financial relationships that could be construed as a potential conflict of interest.
