# Supplementary figures and images for: Disrupted Glutamate Signaling in Drosophila Generates Locomotor Rhythms in Constant Light
Source: Front Physiol. 2020 Mar 6;11:145. doi: 10.3389/fphys.2020.00145 (PMC7069353; doi:10.3389/fphys.2020.00145)

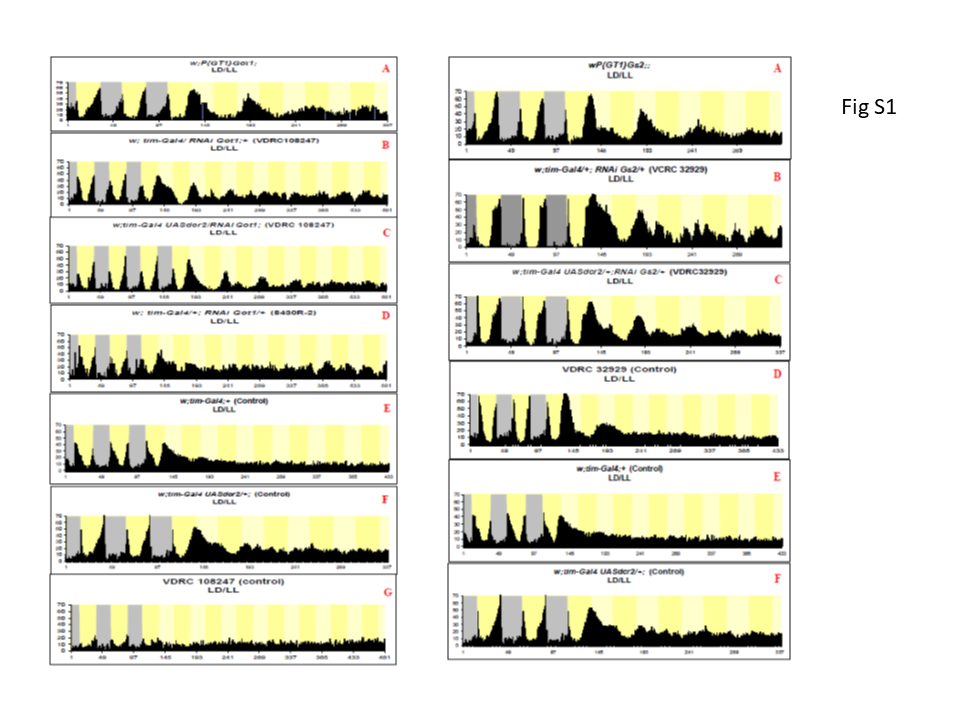

Supplement: Supplementary file 1 [file Image_1.TIF]
