# Supplementary material for: Disrupted Glutamate Signaling in Drosophila Generates Locomotor Rhythms in Constant Light
Source: Front Physiol. 2020 Mar 6;11:145. doi: 10.3389/fphys.2020.00145 (PMC7069353; doi:10.3389/fphys.2020.00145)
Supplement: Supplementary file 2 [file Table_1.DOCX]

Supplementary Table 1

| *Mutants (ID)*  *B Bloomington*  *V VDRC*  *Lk6 kinase* | DD | | | LL | | |
| --- | --- | --- | --- | --- | --- | --- |
|  | % rhythmic | Mean period, h (sem) | N | % rhythmic | Mean period, h (sem) | N |
| *w^1118^;;Lk6^(1)^ /+ (B8706)* | 66.7 | 23.2 (0.24) | 6 | 12.5 | - | 8 |
| *w^1118^;;Lk6^(1)^ /+ (B8707)* | 100 | 23.5 (0.07) | 16 | 18.8 | - | 16 |
| *w;timgal4/+;RNAiLk6/+*  *(V30389)* | 92.5 | 24.1 (0.07) | 40 | 31.8 | - | 22 |
| *w;timgal4/+;RNAiLk6/+*  *(V32885)* | 100 | 23.9 (0.13) | 15 | 28.6 | - | 14 |
| *(V30389)* | 75 | 23.9 (0.25) | 16 | - | - | - |
| *Glycogenin* |  |  |  |  |  |  |
| *w;timgal4/RNAiGlycogenin*  *(V35452)* | 95.6 | 24.0 (0.13) | 23 | 93.7 | 28.9 (0.8) | 16 |
| *(V35452)* | 93.4 | 23.9 (0.17) | 16 | 56.3 | 28.8 (0.57) | 55 |
| *w;timgal4; +* | 71 | 24.5 (0.3) | 31 | 4 | - | 51 |
| *Rab11* |  |  |  |  |  |  |
| *y1 w67c23;;P{wHy}*  *Rab11^DG0950^ /+ (B21463)* | 100 | 23.2 (0.09) | 16 | 31 | - | 16 |
| *w;tim-Gal4/+;RNAi*  *Rab11/;+ (V22198)* | 100 | 24.1 (0.08) | 27 | - | - | - |
| *V 22198* | - | - | - | 20 | - | 10 |
| *katanin80* |  |  |  |  |  |  |
| *y^1^P(SUPor-P)kat80^KG02315^;;*  *(B12979)* | 86.2 | 23.6 (0.19) | 29 | 21.6 | - | 26 |
| *w^1118^ P(EP)kat^80EP620^ (B17187)* | 100 | 23.5 (0.16) | 15 | 0 | - | 15 |
| *w;timgal4/RNAikat80;+ (V24175)* | 85.2 | 24.5 (0.11) | 27 | 58.3 | 28.2 (0.6) | 12 |
| *V24175* | - | - | - | 55 | 28.8 (1.9) | 11 |
| *pebble* |  |  |  |  |  |  |
| *w;;pbl^3^/+ (B9358)* | 100 | 23.6 (0.11) | 16 | 50 | 25.5 (0.9) | 15 |
| *;;pbl^3^/+ (B2452)* | 100 | 23.7 (0.08) | 16 | 12.5 | - | 16 |
| *w;timgal4/RNAipbl;+ (V35349)* | 100 | 24.2 (0.09) | 25 | 14.3 | - | 7 |
| *w;timgal4/RNAipbl;+ (V35350)* | 100 | 24.2 (0.1) | 28 | 78.5 | 26.4 (0.3) | 11 |
| *V35350* | 92.3 | 23.9 (0.14) | 13 | 73 | 26.2 (0.5) | 11 |
| *V35349* | 100 | 23.8 (0.28) | 6 | 36 | - | 11 |

DD and LL rhythmicity of mutants of *LK6 kinase, Glycogenin, Rab11, katanin80 and pebble*

Supplementary Table 2

| *Genotype (ID)*  *V, VDRC* | DD | | |
| --- | --- | --- | --- |
|  | % rhythmic | Mean period, h (sem) | N |
| *w;timgal4/+; RNAiDmGluRA/+ (V1793)* | 100 | 24.1 (0.14) | 28 |
| *w;timgal4/+; RNAiDmGluRA/+ (V1794)* | 100 | 23.8 (0.09) | 31 |
| *w;timgal4/+; RNAiDmGluRA/+ (V103736)* | 100 | 23.7 (0.06) | 32 |
| *w;timgal4/+; RNAiGad1A/+ (V32344)* | 100 | 23.7 (0.09) | 31 |
| *V1793* | 96.7 | 23.9 (0.1) | 30 |
| *V1794* | 100 | 23.8 (0.07) | 32 |
| *V 103736* | 93.8 | 23.4 (0.06) | 32 |
| *V32344* | 96.5 | 23.7 (0.1) | 29 |
| *w; timgal4* | 71 | 24.5 (0.3) | 31 |

Free-running rhythmicity of glutamate receptor (DmGluRA) and glutamic acid decarboxylase (Gad) dsRNAi knockdowns
